# Time-resolved non-invasive metabolomic monitoring of a single cancer spheroid by microfluidic NMR

**DOI:** 10.1038/s41598-020-79693-1

**Published:** 2021-01-08

**Authors:** Bishnubrata Patra, Manvendra Sharma, William Hale, Marcel Utz

**Affiliations:** 1 School of Chemistry, University of Southampton, Southampton, SO17 1BJ UK; 2 Present Address: Department of Chemistry, University of Florida, Gainesville, FL 32611-7200 USA

**Keywords:** Lab-on-a-chip, Microfluidics, NMR spectroscopy, Metabolomics

## Abstract

We present a quantitative study of the metabolic activity of a single spheroid culture of human cancer cells. NMR (nuclear magnetic resonance) spectroscopy is an ideal tool for observation of live systems due to its non-invasive nature. However, limited sensitivity has so far hindered its application in microfluidic culture systems. We have used an optimised micro-NMR platform to observe metabolic changes from a single spheroid. NMR spectra were obtained by directly inserting microfluidic devices containing spheroids ranging from 150 $\upmu$m to 300 $\upmu$m in diameter in 2.5 $\upmu$L of culture medium into a dedicated NMR probe. Metabolite concentrations were found to change linearly with time, with rates approximately proportional to the number of cells in the spheroid. The results demonstrate that quantitative monitoring of a single spheroid with $\le$ 2500 cells is possible. A change in spheroid size by 600 cells leads to a clearly detectable change in the l-Lactic acid production rate ($p=3.5\times 10^{-3}$). The consumption of d-Glucose and production of l-Lactic acid were approximately 2.5 times slower in spheroids compared to monolayer culture of the same number of cells. Moreover, while cells in monolayer culture were found to produce l-Alanine and l-Glutamine, spheroids showed slight consumption in both cases.

## Introduction

Culture of cell clusters (spheroids) provides an intermediate between conventional two dimensional monolayer culture of adherent cells and primary tissue culture^1^. Spheroids model at least some of the three-dimensional aspects of the inter-cellular organisation, as well as 3D transport of nutrients, oxygen, and intercellular signals^2^. They are widely used as models for cancer in both translational and fundamental research^3^. Lab-on-a-chip (LoC) devices allow efficient experimentation with small sample volumes, high throughput, and convenient integration of multiple experimental steps onto a single, compact platform. They are commonly used for the culture of spheroids since they provide detailed control over the conditions of growth.

In most microfluidic studies, information is extracted from the LoC device in a destructive end-point analysis. This can take the form of micro-PCR based genomic or transcriptomic analysis, proteomics by western blot, or an immunoassay targeting a small set of specific biomarkers. In addition, it would be very useful to follow the metabolism of a single spheroid culture longitudinally, i.e., over the course of the entire experiment. This would allow correlating metabolic activity during the culture with the results of the destructive end-point analysis. In-situ metabolic montitoring in LoC devices is possible using electrochemical microsensors^4^, or by LC-MS^5^. However, ampereometric detection requires integration of electrodes into the fluidic system. These are prone to degradation over time, and are in practice limited to a small number of metabolites. LC-MS can detect a very large number of metabolites, but requires removal of aliquots from the chip. NMR spectroscopy is uniquely suited for metabolomic observation of live systems, since it is non-invasive, label-free, and allows for the direct quantitation of analytes. The small sample volumes involved in microfluidic systems, however, pose a challenge in terms of sensitivity. In the case of spheroids, this is exacerbated by the inherently lower metabolic rate compared to monolayer cell culture^6^. LoC devices for spheroid culture need to prevent cell adhesion to the chip surface. This has been accomplished using the air/liquid interface in hanging drops^7–9^, in enclosed chambers with appropriate coatings^10^, and by combining gelation technology with droplet microfluidics^11^. Moreover, spheroids are limited to about 500 $\upmu$m in diameter if necrotic cores are to be avoided, further limiting the number of cells and restricting the absolute metabolic turnover per spheroid. While necrotic cores in spheroids can serve as models of necrotic cancer tissue, they are undesirable in organoids and in cancer spheroid studies that focus on the metabolic activity of viable cells and their response to drugs. Highly efficient NMR micro-detectors have been developed by a number of groups over the past two decades^12–19^ and sensitivities in the vicinity of 1 $\mathrm {nmol}\,\sqrt{\hbox {s}}$ have been achieved with samples around 1 $\upmu$L volume^20^. Our group has recently presented a modular microfluidic NMR system that accommodates generic LoC devices^21^. A detailed characterisation of its performance is given in Ref. ^21^. As we show in the following, this system enables metabolic monitoring of a spheroid of less than 2500 cells.

NMR spectroscopy has been used before to study metabolic differences between spheroid and monolayer cell culture. At the macro scale, Santini et al. have studied MG-63 human osteosarcoma cells grown in spheroids and monolayers and observed the difference in metabolic activity^6^ by $^{1}\hbox {H}$ NMR of cells and perchloric acid cell extracts. The study was limited to a single time point observation, and a very large number of cells was used (order of $10^8$). Real-time monitoring of cells using NMR provides a crucial step towards understanding cellular metabolism. For example, Pilatus et al. have used cells attached on polystyrene microbeads to study oxygen consumption, pH and energy metabolism using NMR spectroscopy^22^. Recently, Wen et al. compared the metabolism of $10^7$ cancer and normal cells in suspension in the presence of an anticancer agent over 2 h^23^. By comparison, the current microfluidic study quantifies the metabolic rates of adherent and spheroid cell cultures for 20 times longer duration on 4 orders of magnitude fewer cells.

While studies on large numbers of spheroids provide averaged information of spheroid metabolism, they cannot capture phenotype variation from one spheroid to another^24^. Spheroids can be difficult to grow in consistent size in large numbers. In some cases, the supply of cells is restricted, for example from patient biopsies. The ability to efficiently quantify the metabolism of single spheroids would open the possibility of massively parallel studies of many spheroids under systematically varied condition such as combinatorial studies of the effects of drugs and culture conditions. Kalfe et al. have demonstrated that it is possible to obtain metabolic information from a large single spheroid (diameter 0.5 mm with 9000 cells)^25^, by combining microfluidic and micro-NMR technology. However, their setup is based on a dedicated fluidic design that provides continuous nutrient supply to the spheroid via evaporation-driven convection^25^. This makes it difficult to derive quantitative metabolic consumption/production rates. It is also not possible to change the experimental modality, for example, to vary the size of the spheroids or to compare with adherent monolayers of the same cell type. More recently, Palma et al. have studied the effect of gamma irradiation on monolayers and spheroids of MCF7 breast cancer cells by NMR spectroscopy of cell suspensions, as well as by micro-NMR imaging of individual spheroids^26^. Localised $^{1}\hbox {H}$ NMR spectra from single spheroids were obtained, but with insufficient signal/noise ratios to allow quantification of metabolites. Clear differences between irradiated and non-irradiated cells could only be detected in much larger cell suspensions (order of $10^6$ cells). Single-spheroid metabolomics has been achieved using mass spectrometry^27^, but as a destructive end-point analysis this approach does not allow longitudinal monitoring of live cultures. In the present contribution, we demonstrate that a flexible microfluidic NMR platform consisting of a modular transmission line NMR micro-probe^21^ in combination with disposable, generic LoC devices makes it possible to quantitatively follow the metabolism of a single spheroid of $< 2500$ mammalian cells (MCF7) in 2.5 $\upmu$L of medium over the course of 48 h. At the same time, the LoC approach provides convenient control over the culture conditions, and can therefore be used to quantitatively compare metabolic data obtained under different experimental modalities. This is exploited to quantitate the metabolic activity of single spheroids as a function of their size, and to compare spheroid to monolayer culture with the same number of cells.

## Results

**Figure 1:**
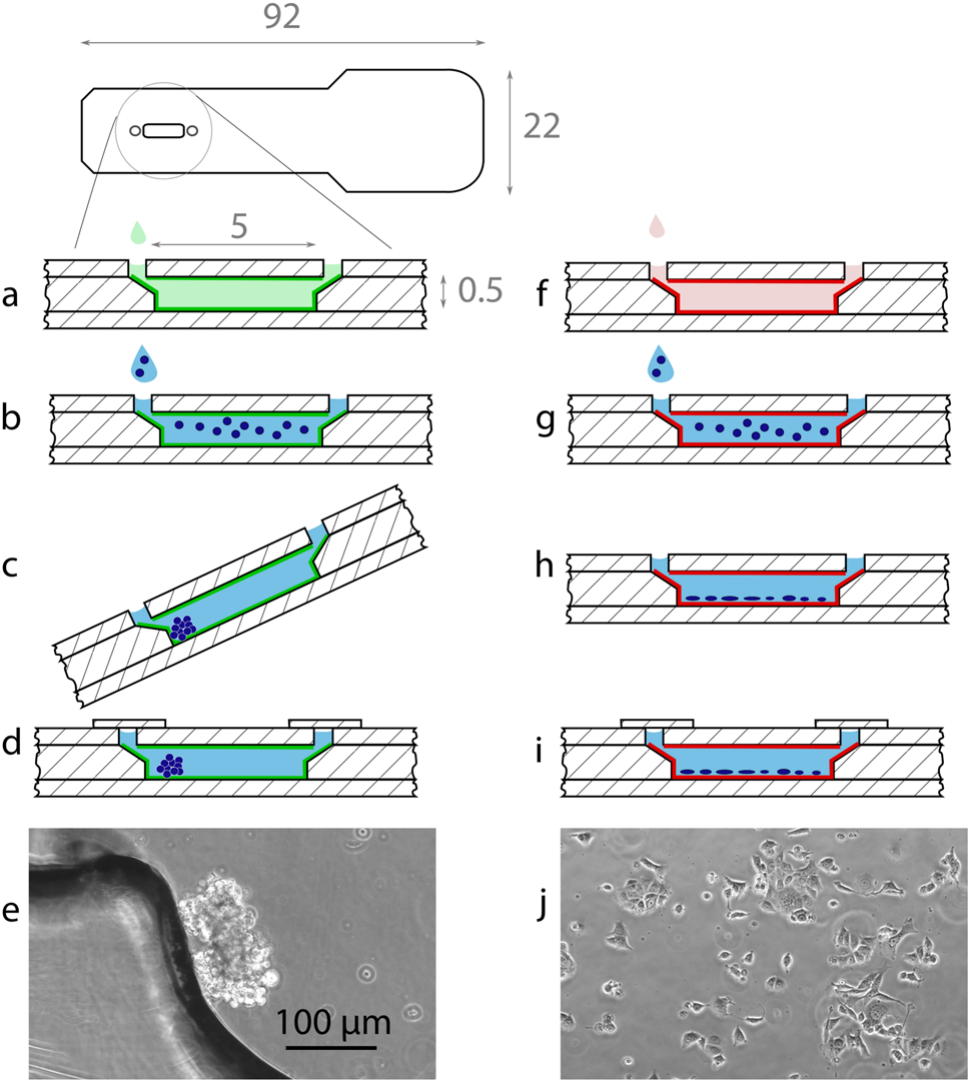
Schematic of the microfluidic device and sample preparation. The device is coated with pluronic F-127 (**a**, dimensions in mm) or fibronectin (**f**). Then, cells are seeded in growth medium (**b,g**), and incubated for 4 h (**c,h**). The pluronic-coated device is held at an angle, allowing the cells to settle at the bottom (**c**). Finally, the devices are sealed with adhesive tape (**d,i**). Phase-contrast micrograph of the spheroid culture (**e**) and adherent monolayer (**j**) of 1800 cells in 2.5 $\upmu$L. (**e,j**) are the same scale.

### Spheroid and adherent layer formation

**Figure 2:**
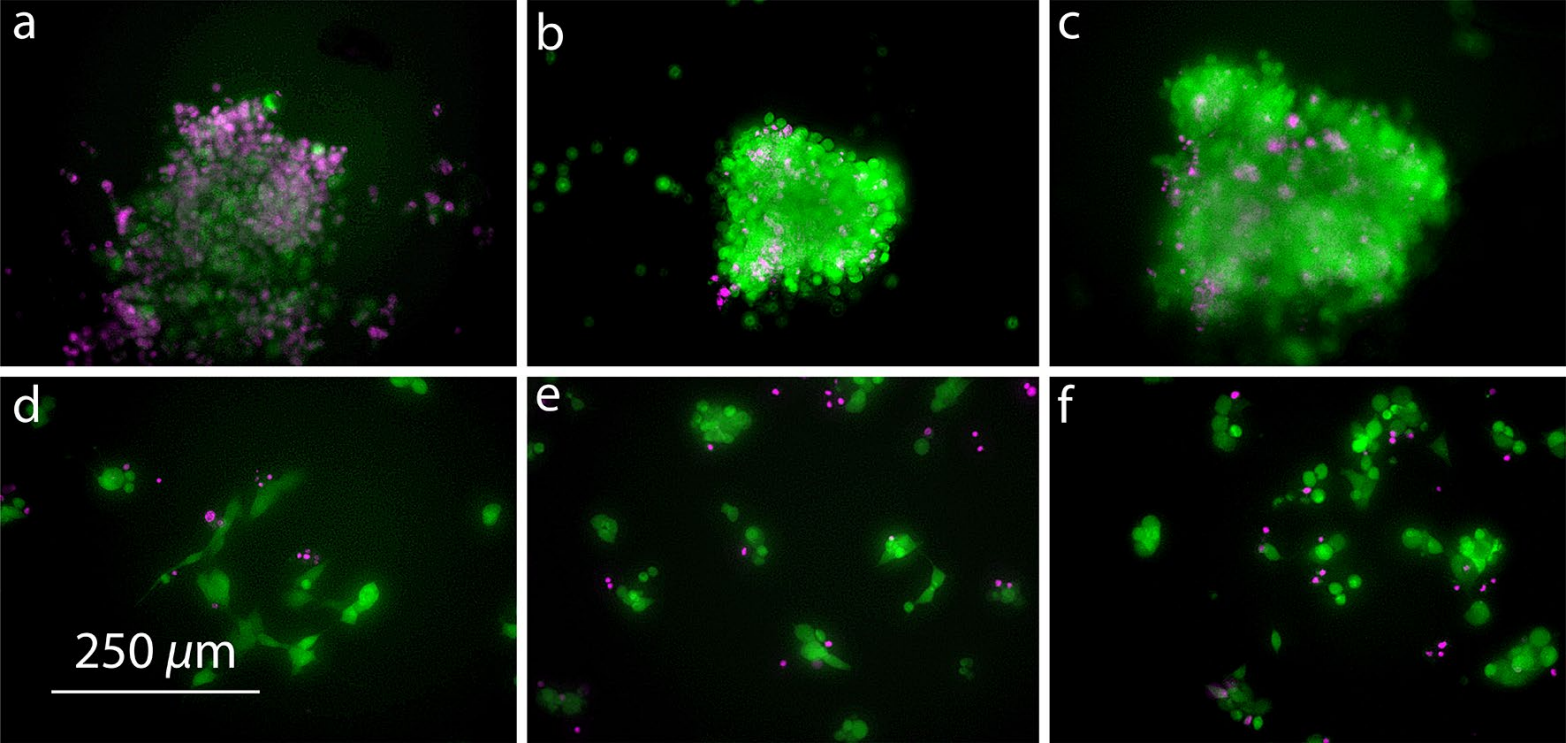
Live(green)/dead(purple) stain of MCF7 cells cultured under hypoxic conditions as a spheroid (**a–c**) and as an adherent monolayer (**d–f**) after 48 h. The seeding number increases from left to right with 1250 cells (**a,d**), 1800 cells (**b,e**), and 2500 cells (**c,f**) in 2.5 $\upmu$L. Scale bar is the same for all the micrographs in this figure.

 LoC devices were internally coated with either pluronic F-127 or with fibronectin (Fig. 1a,f). The former is a polyethylene oxide/poly propylene/polyethylene oxide block copolymer which is well known to prevent protein and cell adhesion^28,29^, while the latter promotes cell adhesion^30,31^. Each device was seeded with a suspension of MCF7 cells in culture medium and incubated for 4 h (Fig. 1b,g). During this time, the pluronic-coated chips were kept at an angle (Fig. 1c), causing the cells to settle near the bottom of the culture chamber (Fig. 1d), while the fibronectin-coated devices were kept lying flat (Fig. 1h,i). This led to the formation of a cell spheroid in the pluronic F-127 coated devices, and an adherent monolayer of cells at less than 20% confluence in the devices coated with fibronectin, respectively, as shown in Fig. 1e,j. The chips were then sealed with an airtight self-adhesive film and were transferred to an incubator. The monolayers were composed of cell clusters, with very few isolated cells (Fig. 1j). Automated image analysis of phase contrast micrographs taken at 4h, 24h, and 48h of culture showed no significant change in cell density or confluency (results given in the SI, Fig. S2), even though slight positive and negative trends (within experimental error) were observed at the lowest and highest seeding densities, respectively (cf SI). Fluorescence micrographs were taken after 48 h of culture, confirming viability of more than 90% (Fig. 2).

### Viability analysis

After 48 h of culture, the viability of the cells in each microfluidic device was assessed using a fluorescent live/dead stain, as shown in Fig. 2. At the lowest seeding density, only a loose spheroid was obtained, and cell viability at the end of the experiment was lower compared to other spheroids (Fig. 2a). The higher seeding densities led to a compact spheroid with only a small number of dead cells present after 48 h (Fig. 2b,c). The monolayer cultures (Fig. 2d–f) show generally good viability, with only a small number of dead cells visible in each case.

**Figure 3:**
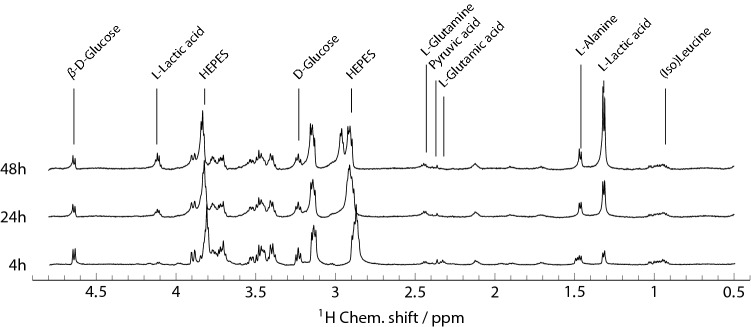
600 MHz $^{1}\hbox {H}$ NMR spectra obtained from a spheroid culture of about 2500 cells (seeding density of 1000 cells/$\upmu$L) after 4 h, 24 h, and 48 h.

### NMR spectroscopy

Figure 3 shows NMR spectra obtained from a spheroid at 4 h, 24 h, and 48 h of culture. The spectra exhibit a resolution of about 3 Hz; the signal/noise ratio is about 200 based on the HEPES buffer peak at 3.82 ppm. The most obvious change over time is the growth of the l-Lactic acid doublet at 1.32 ppm. The same effect is also present in the monolayer culture (data not shown) at a significantly larger magnitude. More subtle features in the spectra reflect changes of the concentrations of other metabolites, as discussed in detail below. It should be noted that the spectra are obtained from the entire culture volume, including both the cells and the surrounding growth medium. The signals are, however, dominated by the surrounding medium, since susceptibility differences lead to broadening of signals from inside the cells^32^.

### pH changes

**Figure 4:**
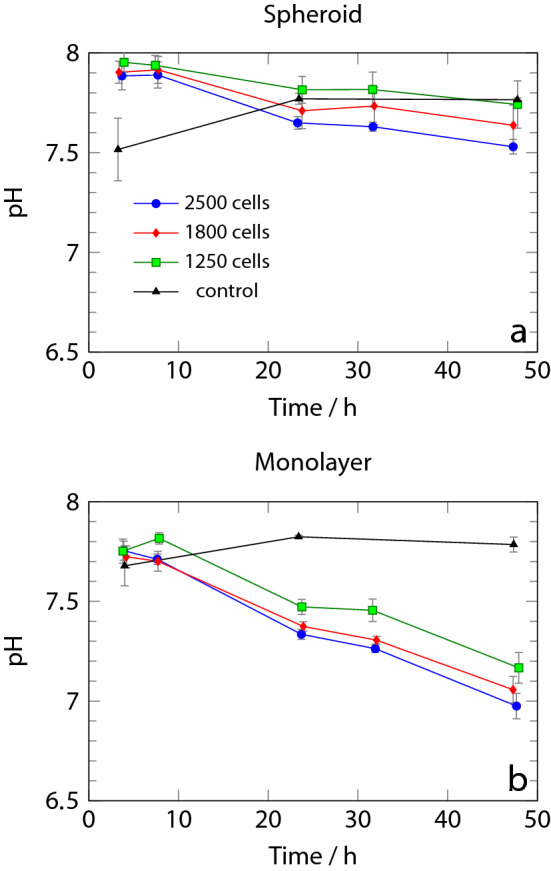
Decrease of pH in the growth medium of culture over 48 h, as obtained from the chemical shift of the HEPES peak near 3.8 ppm **(a)** spheroids; **(b)** monolayer culture. Solid lines are guides to the eye.

 The HEPES buffer signals exhibit gradual shifts which reflect changes in pH^33^. For example, the peak at 2.85 ppm at 4 h of culture in Fig. 3 shifts to 2.95 ppm at 48 h. These shifts can be calibrated to give a quantitative pH reading. Fig. 4 summarises the decrease of the pH in the growth medium, obtained from the HEPES peak at 3.8 ppm (calibration given in the SI, S6). The largest spheroids lead to a drop in pH from 7.9 to 7.6, and a correspondingly smaller effect is observed for the smaller spheroids (Fig. 4a). The effect is more pronounced in monolayer cultures (Fig. 4b).

**Figure 5:**
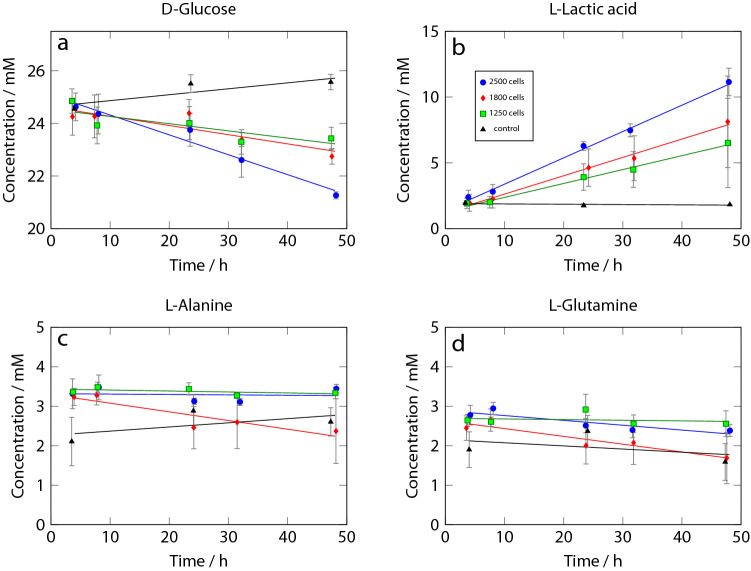
Metabolite concentrations over time in single spheroid culture as a function of time. d-Glucose **(a)**, l-Lactic acid **(b)**, l-Alanine **(c)**, and l-Glutamine **(d)** changes in 2.5 $\upmu$L volume. Error bars represent twice the standard deviation of three separate experiments.

### Metabolite concentrations

More detailed examination of the spectra reveals a range of additional systematic changes over time. These reflect changes in metabolite concentrations in the culture medium (exa-metabolome). In order to quantify metabolite concentrations, the spectra were processed using a model-free approach, which is described in the Methods section.

The HEPES buffer peak at approximately 3.8 ppm was used for normalisation of the spectra, since it has minimal overlap with other peaks. HEPES is not metabolised by the cells. Normalised spectra were then projected onto a set of metabolite reference spectra obtained from the human metabolome database. This yields an intensity value for each metabolite, which is then converted to an absolute concentration using the known d-Glucose concentration in the control experiments as a standard. Fig. 5 shows the evolution of the concentration of several metabolites as a function of time for the different sized spheroids and control experiment without cells. The error bars represent the standard deviation for three separate experiments, which had been carried out over the course of 3 months, and involved newly fabricated chips and new cells each time.

**Figure 6:**
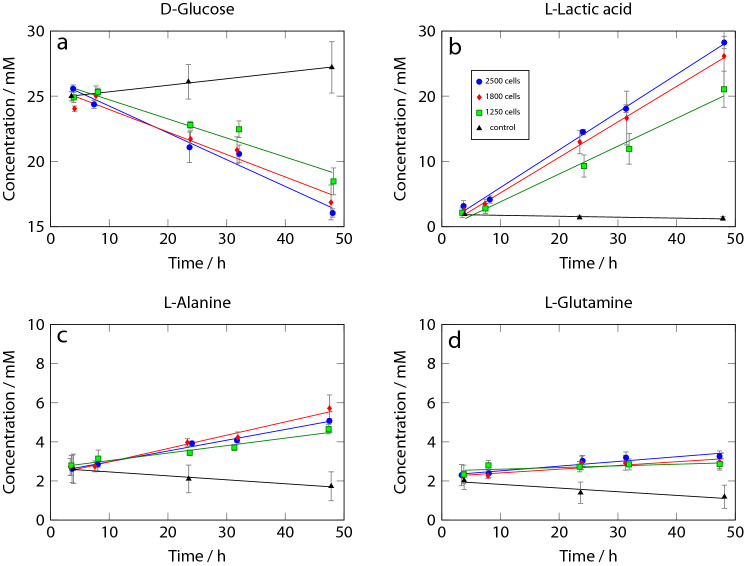
Metabolite concentrations in monolayer culture over time. d-Glucose **(a)**, l-Lactic acid **(b)**, l-Alanine **(c)**, and l-Glutamine **(d)** changes in 2.5 $\upmu$L volume. Error bars represent twice the standard deviation of three separate experiments.

In both the spheroid and monolayer cultures, the concentrations of d-Glucose (Figs. 5a, 6a) and l-Lactic acid (Figs. 5b,  6b) change linearly with time within experimental precision, as shown by the regression lines. The spheroids consume d-Glucose significantly more slowly than the cells in monolayer culture, and their l-Lactic acid production is correspondingly lower by a factor of about 2.5.

While the response of the spheroid and monolayer cultures is similar in the case of d-Glucose and l-Lactate apart from the difference in consumption/production rate, there is a qualitative difference in the cases of l-Alanine and l-Glutamine (Figs. 5, 6c,d). Glutamine and Alanine are present in the medium from the start of the experiment in the form of Glutamax. While spheroids do not seem to add detectable amounts of l-Alanine (Fig. 5c), there is a clear linear increase in the l-Alanine concentration from 2 to 5 mM in the monolayer culture over 44 h (Fig. 6c). The monolayer cells also seem to produce l-Glutamine (Fig. 6d), while the l-Glutamine concentration shows a slightly decreasing trend in the case of the spheroid culture (Fig. 6d). It is known that MCF7 cells can produce l-Glutamine^34^, and it has been speculated that hypoxic conditions lead to upregulation of l-Glutamine synthetase and downregulation of glutaminases^26^. Our results suggest that while this effect is visible in the monolayer culture, it is largely absent in the cell spheroids.

**Figure 7:**
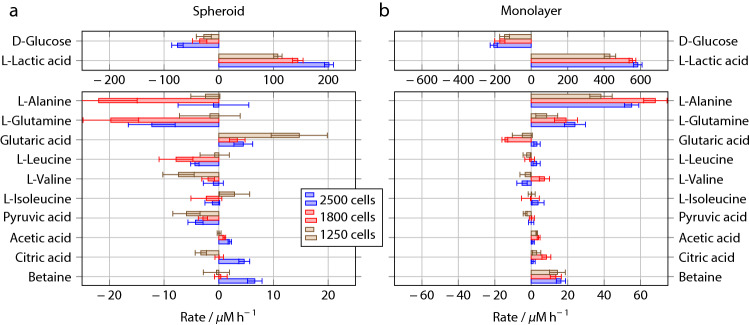
Production (positive) or consumption (negative) rates of different metabolites as determined from the NMR spectra for a single spheroid **(a)** and monolayer culture **(b)**. Note that the scale for the spheroid results is expanded threefold compared to the monolayer data. Error bars represent the standard deviation from three separate experiments.

**Figure 8:**
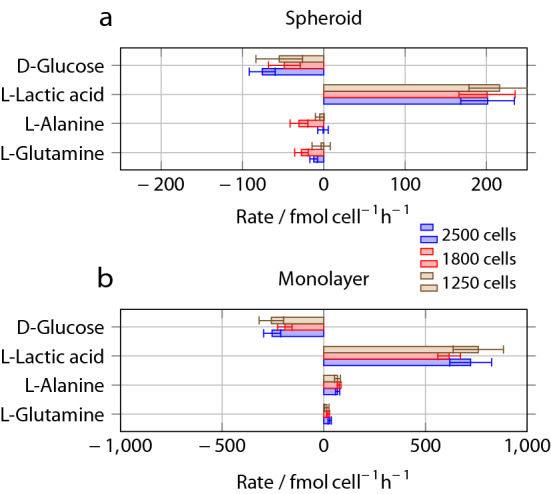
Production (positive) or consumption (negative) rates per cell, obtained from the NMR spectra for a single spheroid **(a)** and monolayer culture **(b)**. The scale for the spheroid results is expanded by a factor of 4 compared to the monolayer data. Error bars represent the standard deviation from three separate experiments, and take into account the errors in cell numbers as well as fabrication tolerance error in the sample volume.

### Rates of change

 The full set of data obtained can be visualised in terms of the rates of change in metabolite concentrations, as shown in Fig. 7. Results from experiments at all three seeding densities are shown here. The l-Lactic acid production rate clearly changes with the cell number in spheroids changing from 100 $\upmu \mathrm {M\,h^{-1}}$ in the smallest spheroid ($1250\pm 185$ cells) to 200 $\upmu \mathrm {M\,h^{-1}}$ in the largest spheroid ($2500\pm 375$ cells). These differences are statistically significant with *p*-values of $1.2\times 10^{-3}$ for the adherent cells and $1\times 10^{-4}$ for the spheroids, respectively (full information on statistical analysis is given in the SI). Even the intermediate spheroid ($1800\pm 270$ cells) can be distinguished from the other two spheroids without the error bars overlapping. A corresponding trend can be seen in the d-Glucose consumption rates, which range from $-27\pm 13\;\upmu \mathrm {M\,h^{-1}}$ for the smallest to $-76\pm 11\;\upmu \mathrm {M\,h^{-1}}$ for the largest spheroids ($p=4.2\times 10^{-3}$). The d-Glucose consumption and l-Lactic acid production rates in the monolayer cultures show a similar trend, but are consistently a factor of 2.5–3 larger than those in the spheroids. It is also clear that the monolayer cells produce significant amounts of l-Alanine and l-Glutamine, while the corresponding rates are either zero within experimental error or negative for the spheriods.

## Discussion

To our knowledge, this is the first quantitative determination of the metabolic rate of an individual cell spheroid with less than 2500 cells. The present approach can distinguish the l-Lactic acid production rates of two spheroids differing by 600 cells in size ($p=3.5\times 10^{-3}$). The repeatability of the NMR-derived concentration data is reflected in the error bars shown in Figs. 5 and  6. The concentrations derived from the spectra are somewhat affected by random spectral noise, as well as systematic differences between spectra obtained on different days and from different LoCs due to imperfections in shimming and water suppression. Some of these artefacts could be reduced by manual processing of all spectra and optimisation of phase adjustments and baseline corrections. However, we decided to fully automate the analysis to more closely reflect its reliability under realistic conditions.

Individual spheroids have been studied by micro-NMR imaging and spectroscopy by Palma et al.^26^. They obtained localised NMR spectra from a spheroid. The resulting signal/noise ratio was, however, not sufficient to quantify metabolite concentrations. Our microfluidic study measures the NMR spectrum of the spheroid and the culture medium, and is thereby able to provide quantitative information on metabolite consumption and production rates from a single spheroid over an extended period of time.

As already mentioned, Kalfe et al. have presented a microfluidic NMR study of a spheroid of approximately 9000 cells^25^. However, the limits of quantification and detection are difficult to assess, as no systematic error analysis is given in their paper. The setup presented here provides flexible control over the experimental conditions. This is exemplified in the ability to perform a control experiment with an adherent monolayer culture, simply by changing the surface coating of the chip. Other comparison experiments can be easily conceived, for example culture under active perfusion, modulation of oxygen supply, or changes in temperature.

Compared to adherent monolayer culture at the same seeding density, the d-Glucose consumption and l-Lactic acid production rates of the spheroids are a factor of 2.5 to 3 lower (Fig. 7a). This could be either due to a smaller number of active (live) cells in the spheroids, or due to inherently different behaviour of the cells in spheroid and in monolayer culture. Fig. 8 shows the most important metabolic rates on a per-cell basis; the data has been obtained by dividing the concentration rates in Fig. 7 by the number of cells. In the case of the spheroids, the cell number is the nominal number based on the seeding density, while in the case of the monolayer data, results from cell counting by image analysis have been used. Within experimental error, the metabolic rates for d-Glucose and l-Lactic acid *per cell* are independent of spheroid size. The fraction of cells that sit on the surface of the spheroid, and therefore have direct access to the medium, is expected to decrease with spheroid size. If there were a drastic difference between the metabolic rates of the cells on the surface and those in the core of the spheroids, one would expect to find different average metabolic rates per cell depending on the spheroid size. Our results therefore suggest that the metabolic activity of the cells in the spheroids is quite evenly distributed. While spheroids significantly larger than the ones studied here are known to produce necrotic cores^35^, flow cytometry studies have shown that spheroids of similar and even somewhat larger size do not^36,37^. We therefore conclude that the lower metabolic rate of the spheroids is indicative of inherently different behaviour of the cells, rather than simply reflecting reduced participation. This is also corroborated by the qualitative difference in the cases of l-Alanine and l-Glutamine.

The ratio between the rate of l-Lactic acid production and d-Glucose consumption is more than 2 in all cases (cf. Fig. 7). However, Stoichiometry would dictate that each molecule of d-Glucose can be converted at most into two molecules of l-Lactic acid. This surprising finding is not due to measurement error. Determining concentrations from integrated NMR peak intensities is well established. Under the experimental conditions used here, differences between spin-lattice relaxation times of different compounds and between different peaks of the same compound could potentially influence the results. Measurements (SI) have shown that under the present conditions, these effects are minimal, with all spin-lattice relaxation times for both glucose and lactic acid well below the repetition time of 3s. Indeed, a direct calibration experiment with a sample of known d-Glucose and l-Lactic acid concentration (SI) showed that the d-Glucose/l-Lactic acid concentration ratio obtained by NMR is accurate to within 10%. At this point, we do not have an explanation for this surprising phenomenon. Possibly, there is another source for the excess amount of l-Lactic acid which is not visible to the liquid NMR measurement, for example, d-Glucose contained a priori within the cells.

The metabolic activity of MCF7 cells in monolayer culture has been determined as a function of oxygen partial pressure in a macroscopic experiment ($>10^6$ cells) using radiotracers by Guppy et al^38^. They found that the l-Lactic acid production rate falls steeply with oxygen concentration; they obtained a value of 250 $\mathrm {fmol\,h^{-1}}$ per cell at atmospheric oxygen (203 $\upmu \mathrm {M}$), 800 $\mathrm {fmol\,h^{-1}}$ per cell at 5 mM oxygen and just over 1000 $\mathrm {fmol\,h^{-1}}$ per cell at 0 $\upmu$M oxygen. The value obtained here is $720\pm 100$ $\mathrm {fmol\,h^{-1}}$ per cell, which may be due to oxygen leakage into the chip resulting in a residual oxygen concentration somewhat above 5 $\upmu$M.

In summary, this is the first on-chip spheroid formation and quantitative metabolomics study via NMR. The above results further show that NMR in combination with microfluidics can provide a flexible platform for the quantitative and non-invasive study of the metabolism of a single spheroid culture. Consumption/production rates of the most abundant metabolites can be determined accurately from $\le$2500 cells. For MCF7 cells, the metabolic rates of d-Glucose and l-Lactic acid are at least 2.5 times lower in spheroids than in monolayer culture.

## Methods

### Device design and fabrication

 Microfluidic devices were fabricated using cell cast PMMA (poly(methyl methacrylate)) sheets (Weatherall Equipment, Wendover, UK) with a culture chamber of 2.5 $\upmu$L volume. The devices were designed using AutoCAD 2016 and consist of 3 PMMA sheets. The sheet thickness was 200 $\upmu$m for both the top and the bottom layers and 500 $\upmu$m for the middle layer. PMMA sheets were cut to desired size and functionality (holes or channels) using a laser engraving system (L3040 from HPC Laser LTD, Elland, UK). The sheets were bonded using the protocol described in^39^. Briefly, the cut sheets were washed with isopropanol (Sigma-Aldrich); ethanol (Sigma-Aldrich); and isopropanol again. The sheets were air-dried and the bonding surfaces were exposed to oxygen plasma for 80 secs using a Bd-20AC laboratory corona treater (Electro-Technic Products, Chicago, IL, USA). 2.5% of dibutyl phthalate (DBP) (Sigma-Aldrich) in isopropanol was used as a plasticiser. A single coat of DBP solution of 18 $\upmu$L was applied to bonding surfaces. PMMA sheets were kept in an oven at 65$^{\circ }$C for 15 minutes before bonding them under 20 MPa of pressure at 85$^{\circ }$C for 15 minutes using a hydraulic press (Specac Ltd, Kent, United Kingdom).

### Cell culture and spheroid formation

 MCF7 cells from ATCC, UK, were used in this research^40^. Stock cells, within 5–15 passage number, were cultured with Dulbecco’s Modified Eagle Medium (1×) + GlutaMAX (Gibco, UK, 31966-021) with 10% Fetal Bovine Serum (Gibco) and 1% Amphotericin B (Sigma-Aldrich, UK) using a T-75 Flask (Thermo Fisher Scientific, UK) at 37$^{\circ }$C and 5% CO$_2$. Microfluidic devices, after fabrication as described above, were sterilised with UV and Virkon solution for 30 minutes. The devices were pre-coated overnight with pluronic F-127 (Sigma-Aldrich) [10 mg/ml in DI] for spheroid culture. Pluronic F-127 is a polyethylene oxide / poly propylene / polyethylene oxide block copolymer which is well known to prevent protein and cell adhesion cell attachment to the surface^28,29^. A cell suspension of 2.5 $\upmu$L with culture medium + 25 mM HEPES buffer (Gibco) was introduced into 3 devices. We used three different cell densities 500; 750; and 1000 cells/$\upmu$L. A device precoated with pluronic with no cells but culture medium + 25 mM HEPES was used for a control experiment. The devices were put inside a cell culture incubator using a humidified box. During the culture, the devices were kept at an angle to collect the cells to form a spheroid. HEPES provides extra buffering capacity at the absence of the supply the CO$_2$ in the cell culture medium^41^. Bovine Plasma Fibronectin (Gibco) [1 mg/ml in 20 mM sodium dibasic phosphate] was used as precoating for monolayer cultures with the same cell seeding density in parallel. Fibronectin is known to promote cell attachment^30,31,42^. A device precoated with fibronectin with no cells but culture medium + 25 mM HEPES was used for control experiment.

Within 4 h, cells aggregated to form a spheroid or attached to the device surface to form a monolayer. The devices were sealed at both inlet and outlet using optical adhesive tape at this point [MicroAmp optical Adhesive Film, Applied Bioscience, USA]. For the subsequent 44 h of culture, oxygen supply was restricted, resulting in hypoxic conditions. During this time the chips were periodically removed from the incubator and inserted into an NMR spectrometer (for 15 minutes) to obtain the NMR data.

### NMR acquisition and processing

$^1\mathrm {H}$ NMR spectra and micrographs were taken from each chip at 4 h, 8 h, 24 h, 32 h, and 48 h after cell seeding; a selection of the micrographs is shown in the SI. In addition, controls without cells were run at the same time points. The entire experiment was run three times with newly made microfluidic devices, resulting in a collection of more than 100 individual NMR spectra.

$^1$H NMR spectra were acquired using a Varian VNMR 600 MHz spectrometer with premium shielded 14.1 T magnet, and a custom made transmission-line probe that can accommodate planar microfluidic devices of approximately 90 mm $\times$ 20 mm $\times$ 1 mm dimension ($l\times w\times h$). The sensitive region of the probe accommodates a sample chamber of 5 mm length, 1 mm width, and 0.5 mm thickness, corresponding to a measurement volume of 2.5 $\upmu$L. Spectra were acquired using a $T_2$ filter to suppress macromolecular contributions to the signal. The PROJECT sequence ^43^ was used, which refocuses evolution under the *J*-couplings and leads to pure absorption multiplets. The length of a $\pi /2$ pulse was 3.2 $\upmu$s. 16k points were acquired over a spectral width of 12 kHz (20 ppm). Water signal was suppressed by continuous-wave pre-saturation for 2s at a nutation frequency of 200 Hz. 256 transients were averaged with a repetition delay of 3 s. Free induction decays were Fourier transformed on 32k points with Lorentzian line broadening of 1 Hz. Zero and first order phase correction was automatically performed using the entropy minimisation algorithm by Chen et al.^44^. The baseline of the real part of the resulting spectrum was then corrected using the algorithm by Golotvin et al^45^. The chemical shift axis of each spectrum was adjusted such as to centre the l-Lactic acid doublet at 1.317 ppm. All processing was done using home-written scripts in Julia^46^.

### NMR data analysis and deconvolution

Metabolites were identified by detecting peaks in the spectra, and comparing the peak positions to all NMR peak lists available in the human metabolome database. Peaks were identified by first finding the positions of local maxima in the magnitude spectra. Each candidate maximum was then locally fitted in the phased spectrum with a Lorentzian function. This resulted in a list of peak intensities, widths, and chemical shift positions. For each metabolite with a suitable reference spectrum in the HMDB, a match score was calculated by adding the intensity of the reference peaks (which are normalised in the HMDB such that the sum of all peak intensities is unity) with a positive sign if they were present in the measured spectrum, and a negative one if they were not. The metabolites were then listed in decreasing order of score. An example of such a list is shown in the SI.

12 metabolites that appeared consistently in the top 25 highest scores in the cell culture spectra were selected for quantitative analysis. These were: d-Glucose, l-Lactic acid, l-Alanine, l-Glutamine, Glutaric acid, l-Leucine, l-Valine, l-Isoleucine, Pyruvic acid, Acetic acid, Citric acid, and Betaine. For each of these metabolites, the number of protons that contribute to the NMR spectra was determined. For example, d-Glucose contains 12 protons, but the hydroxyl ones are invisible in the NMR spectra under the present conditions due to saturation transfer from water; for this reason, the relevant number of visible protons in d-Glucose is 6. Using this information together with the peak lists from the HMDB (cf SI, Table S1), a reference spectrum with Lorentzian signals of appropriate intensity was calculated on the exact same digital grid as the experimental spectra. These quantitative reference spectra (QRS) are normalised such that they all correspond to the same concentration. The QRS are then arranged in a $(m\times n)$ matrix $\mathbf {Q}$, where *m* is the number of data points in the spectra (typically, 32k), and *n* is the number of metabolites in the set (typically, 12). Concentrations were extracted from the experimental spectra as

$$\begin{aligned} \tilde{\mathbf {c}} = \mathbf {Q}^+\mathbf {s}, \end{aligned}$$

where $\mathbf {s}$ is the $m\times 1$ vector containing the experimental spectrum, and $\mathbf {Q}^+$ is the Moore–Penrose inverse (computed by singular value decomposition) of $\mathbf {Q}$. The components of the $n\times 1$ vector $\tilde{\mathbf {c}}$ are the resulting un-normalised metabolite concentrations, which are the concentrations up to a common calibration factor $\alpha$. This was determined from the known concentration of Glucose in the control spectra. The concentrations $\mathbf {c}$ were therefore obtained as

$$\begin{aligned} \mathbf {c} = \alpha \mathbf {Q}^+\mathbf {s}. \end{aligned}$$

The QRS are shown for a set of metabolites in the SI, Fig. S3, along with the experimental spectrum, and a fit computed from the un-normalised concentrations $\tilde{\mathbf {c}}$ as

$$\mathbf {s}_{\mathrm{fit}} = \mathbf {Q}\tilde{\mathbf {c}}.$$

### Viability analysis after the NMR experiments

 After the NMR experiments, cell viability was analysed on the same devices using Live/dead cell imaging kit [life technologies, Eugene, OR]. A 2.5 $\upmu$L solution of a 2× stock was directly introduced into each device. Each device was imaged with excitation wavelength 470 nm (for live stain) and 535 nm for (for dead stain) using a Nikon-Ti5 microscope and Nikon 20× objective (NA: 0.45) after 15 min incubation at room temperature. Images were analysed using ImageJ software.

## Supplementary information

Supplementary Information.
