# Supplementary material for: Time-resolved non-invasive metabolomic monitoring of a single cancer spheroid by microfluidic NMR
Source: Sci Rep. 2021 Jan 8;11:53. doi: 10.1038/s41598-020-79693-1 (PMC7794408; doi:10.1038/s41598-020-79693-1)
Supplement: Supplementary file 1 — Supplementary Information. [file 41598_2020_79693_MOESM1_ESM.pdf]

Supporting Information: Time-Resolved Non-Invasive Metabolomic Monitoring of a Single Cancer Spheroid by Microfluidic NMR

Bishnubrata Patra, Manvendra Sharma, William Hale, and Marcel Utz*

School of Chemistry, University of Southampton, United Kingdom SO17 1BJ

December 1, 2020

Contents

1 Microscopic imaging and cell counting 1

2 Calibration Experiments 2

2.1 Spin-Lattice Relaxation Measurements 2

2.2 D-Glucose/L-Lactic acid Ratio Calibration 6

2.3 HEPES chemical shift as pH indicator 6

2.4 Statistical Analysis of Metabolic Rates 7

1 Microscopic imaging and cell counting

Each microfluidic device is followed with phase-contrast imaging after the cell seeding in a time-lapse manner. Fig. S1 shows the time evolution of the spheroids with different cell seeding numbers. However, the spheroids were not fixed at a particular position of the chip during the whole 48 hours of culture. The spheroid size has been determined for cell seeding number 1800 and 2500 at 48 hours from three independent experiments and found to be 205 ±16 μm and 273

*Corresponding Author, `marcel.utz@gmx.net`

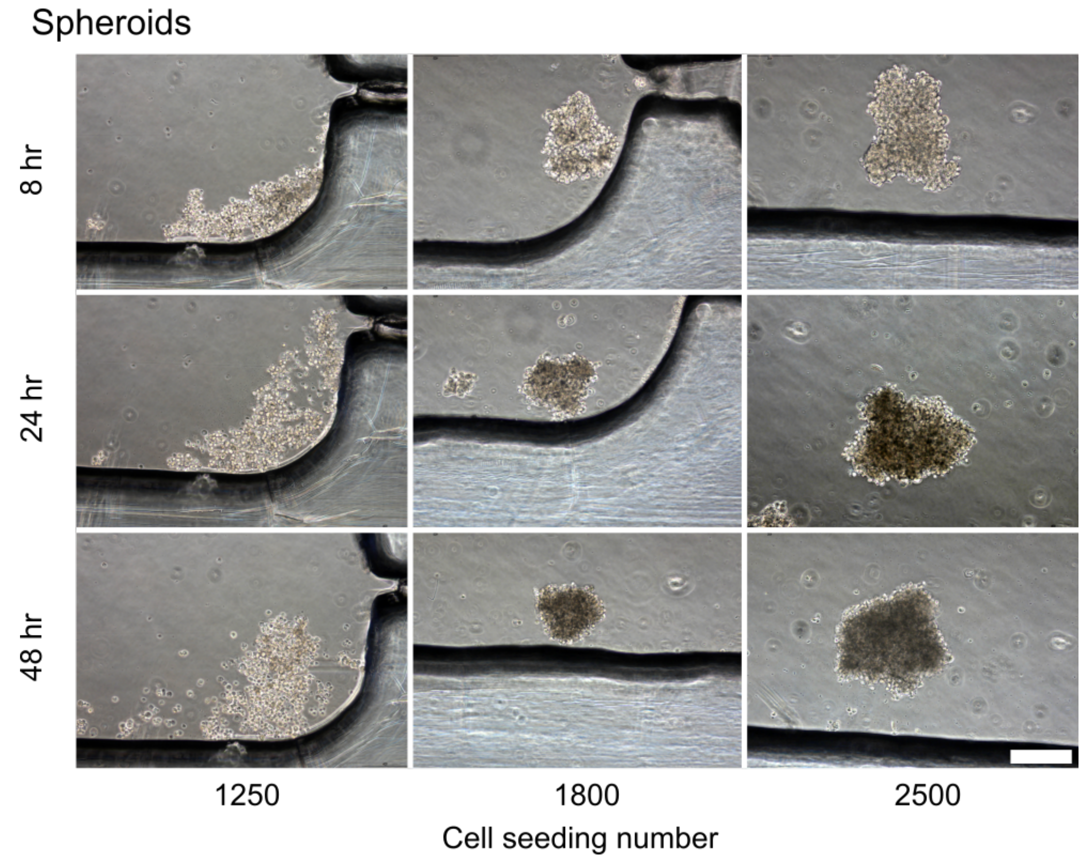

Figure S1: Time-lapse monitoring of a single spheroid of MCF-7 cells formed and culture inside an NMR compatible microfluidic device. Scale bar 200 μm.

$\pm$ 59 μm. The spheroid size was not determined for cell seeding number 1250 as it did not form a compact spheroid. We analyse the cell number of a frame at 4h, 24h and 48h after seeding with an automated program using ImageJ Software (Fig. S2). The total number of the cells was estimated by the ratio of frame area and total area for the attached cell culture. Average cell number was estimated from three independent sets of cell culture with the same seeding density.

2 Calibration Experiments

2.1 Spin-Lattice Relaxation Measurements

To ensure that the concentrations determined from the NMR spectra are not affected by variations in the spin-lattice relaxation times between different metabolites and/or different spectral lines, an inversion recovery experiment was carried out on fresh growth medium. This experiment was performed on a Bruker 600 MHz Avance Neo spectrometer with a Bruker 5 mm BPO

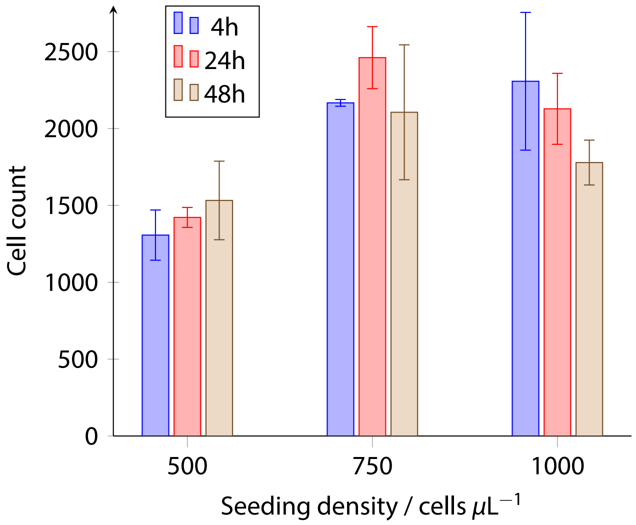

Figure S2: Counts of attached cells in monolayer cultures obtained by automated processing of phase contrast micrographs.

Table S1: List of 1H NMR reference spectra entries in the Human Metabolome Database (hmdb.ca) used in the present work. The accession number refers to the unique entry in hmdb.ca for each metabolite, whereas the spect_id identifies the corresponding spectra. For a complete list of references to these data refer directly to HMDB.ca

accession	name	solvent	spect_id
HMDB0000122	D-Glucose	Water	1093
HMDB0000190	L-Lactic acid	Water	1162
HMDB0000161	L-Alanine	Water	1120
HMDB0000641	L-Glutamine	Water	1452
HMDB0000661	Glutaric acid	Water	1461
HMDB0000687	L-Leucine	Water	1477
HMDB0000883	L-Valine	Water	1582
HMDB0000172	L-Isoleucine	Water	1136
HMDB0000243	Pyruvic acid	Water	1266
HMDB0000042	Acetic acid	Water	1048
HMDB0000094	Citric acid	Water	1080
HMDB0000043	Betaine	Water	1049

Table S2: Example of a score list of metabolites obtained by matching reference peak lists in the human metabolome database to identified peaks in the spectrum of a microfluidic monolayer cell culture.

```
Overall Matches
===============
     HMDB ID SpectID Name                                 Score

 HMDB0000122    1093 D-Glucose                            16.07
 HMDB0003345    1953 Alpha-D-Glucose                      13.60
 HMDB0000641    1452 L-Glutamine                          10.36
 HMDB0003418    1967 D-Tagatose                            9.51
 HMDB0003423    1968 D-Glutamine                           8.12
 HMDB0002467    1893 A-Ketoglutaric acid oxime             7.15
 HMDB0011632    2050 L-Iditol                              5.14
 HMDB0000828    1559 Ureidosuccinic acid                   4.25
 HMDB0000765    1531 Mannitol                              4.13
 HMDB0000149    1110 Ethanolamine                          4.09
 HMDB0000251    1277 Taurine                               3.98
 HMDB0000883    1582 L-Valine                              3.97
 HMDB0000557    1419 L-Alloisoleucine                      3.71
 ...
```

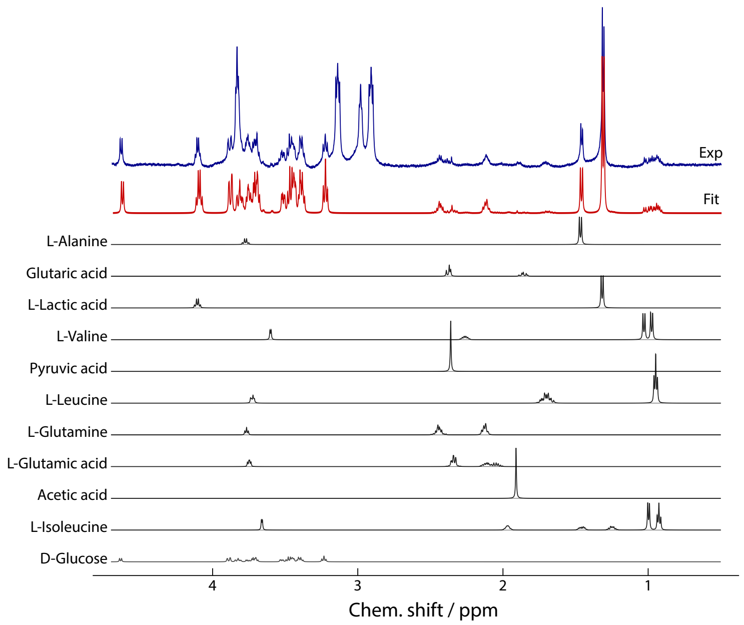

Figure S3: Decomposition of experimental proton NMR spectrum (top trace) into components. The resulting fit is shown in red. The individual metabolite spectra are quantitative reference spectra (QRS), normalised to the same concentration. The fitted spectrum is the weighted sum of these QRS calculated as described in the NMR Data analysis and deconvolution of the methods section of the manuscript. The HEPES signals are omitted from the deconvolution because of their chemical shift variation due to the pH change.

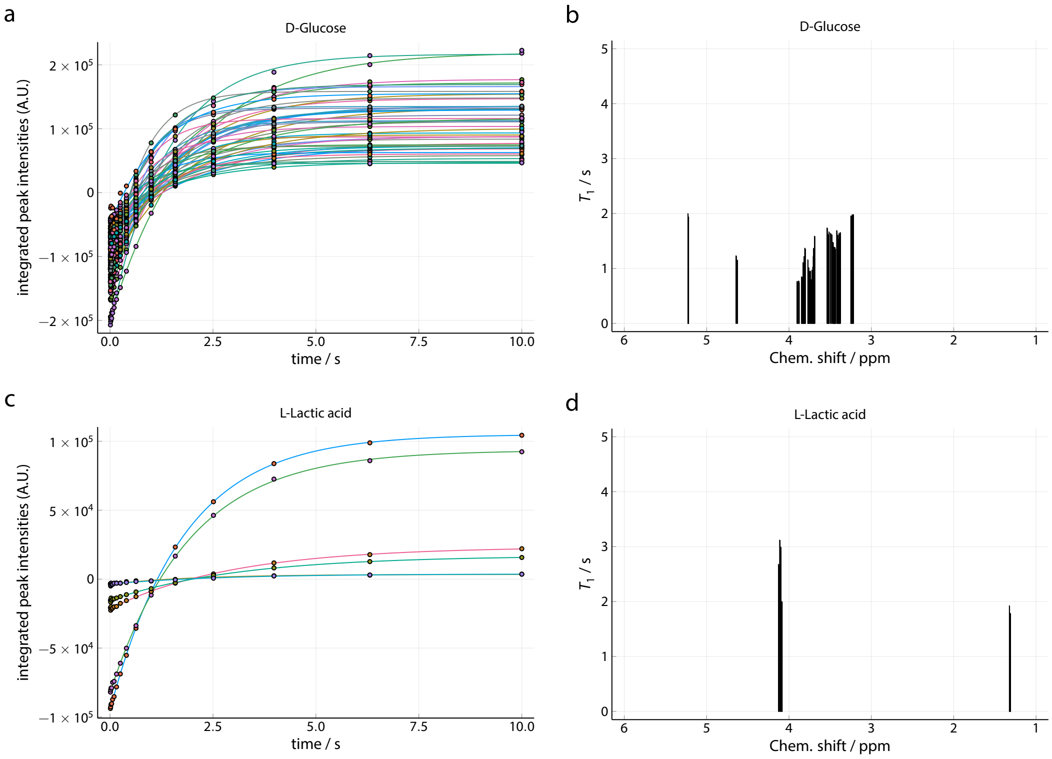

Figure S4: T_1 inversion recovery measurement of D-Glucose and L-Lactic acid in a sample of DMEM medium in a 5 mm NMR sample tube at 600 MHz. a: The dots are integrated peak intensities in arbitrary units of D-Glucose peaks as a function of recovery delay. The different color lines are the exponential fits for different peaks. b: T_1 values obtained from the fits shown in a; each bar is shown at the respective chemical shift of the corresponding resonance line, and the bar height indicates the T_1 value. Similar to a and b, c and d show the integrated peak intensities and exponential fits for different L-Lactic acid peaks and, the T_1 values respectively.

probe. Some of the results are shown in Fig. S4. Of particular interest were the relaxation times of D-Glucose and L-Lactic acid, due to the surprising finding of the L-Lactic acid production rate exceeding twice the D-Glucose consumption rate. This observation immediately led to the suspicion that the relatively short relaxation delay of 3s that was used in the acquisition of the NMR spectra would depress the D-Glucose signals due to incomplete T_1 relaxation between scans. However, the measured T_1 values of D-Glucose, with some variation over the different signals, are all below 2s. The ones for L-Lactic acid (Fig. S4(d)) are just under 2s in the case of the methyl doublet at 1.17 ppm; the quartet at 4.1 ppm is nearer 3s. From this, one would expect the L-Lactic acid/D-Glucose ratio to be slightly *underestimated* rather than overestimated by the NMR protocol employed in this study.

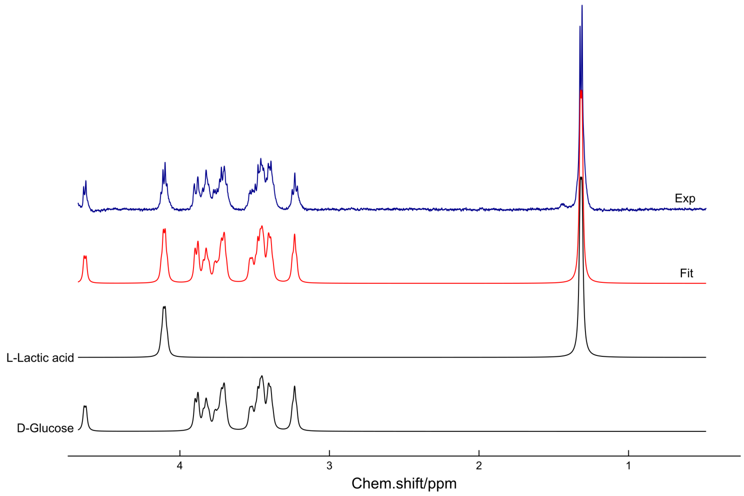

Figure S5: Spectrum acquired from a solution of glucose and lactic acid (both 20 mM) dissolved in water using same pulse sequence and parameters as cell experiments (Top). Calculated traces for L-Lactic acid (middle) and D-Glucose (bottom) by the processing script using data from HMDB database.

2.2 D-Glucose/L-Lactic acid Ratio Calibration

The validity of the NMR pulse sequence and data analysis process for extracting the metabolite concentrations was verified using a solution of D-Glucose and L-Lactic acid dissolved in water at the same concentration. First 100 μL of 200 mM D-Glucose and L-Lactic acid solutions each was dissolved in 700 μL of water. pH was then adjusted to 7.4 by gradually adding $\sim$50-100 μL of 1M NaOH solution to obtain a solution of 1000 μL with exact same concentration of D-Glucose and L-Lactic acid ($\sim$20 mM). The modular microNMR system can be adapted easily to different field strengths. The verification experiments were performed at 11.7 T magnet using the same pulse sequence and parameters in a same type of device as used for the cell experiments. Fig. S5 shows the NMR spectrum from the sample, the fit and the calculated traces by the script for L-Lactic acid and D-Glucose. The relative concentration of D-Glucose and L-Lactic acid was calculated to be within 10% by the script.

2.3 HEPES chemical shift as pH indicator

The chemical shift of the HEPES buffer signal changes due to the change in the pH. The chemical shift of the HEPES signal was measured for a sample of DMEM culture medium with 25 mM HEPES at know pH values. pH of the solution was adjusted by adding HCl or NaOH.

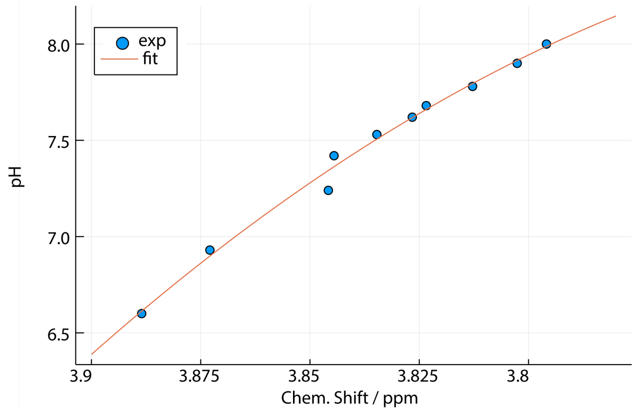

Figure S6: Calibration curve for the pH-dependence of the chemical shift of the HEPES buffer signal at approximately 3.8 ppm.

Chemical shift position of the HEPES signal at different pH is shown in Fig. S6. The chemical shift of HEPES peak in the culture experiments was used to estimate the pH at different time points during the experiment. The experimental data was interpolated (solid line in Fig. S6 by a quadratic function as

$$\mathrm{pH} = -603.79 + 332.99\,\delta - 45.266\,\delta^2, \tag{S1}$$

where δ is the observed chemical shift in ppm of the HEPES peak near 3.8 ppm.

2.4 Statistical Analysis of Metabolic Rates

To assess the statistical signifiance of differences between any pair of the metabolic rates $R_1 > R_2$ reported in Figures 7 and 8, the data were subjected to Student's t-test, with the null hypothesis that $R_1 \leq R_2$. The resulting t statistic was calculated as

$$t = \frac{\bar{R}_1 - \bar{R}_2}{\sqrt{s_p^2 \frac{2}{n}}}, \tag{S2}$$

where $\bar{R}_1$ and $\bar{R}_2$ are the experimental averages of the two rates in question, n is the number of samples in each set (in the present case, $n = 3$), and s_p is the pooled standard deviation

$$s_p = \sqrt{\frac{s_{R_1}^2 + s_{R_2}^2}{2}}, \tag{S3}$$

with the individual standard deviations (obtained from the quality of the linear fits as described in the main manuscript) s_{R_1} and s_{R_2}, respectively. The number of degrees of freedom used for

the t-test is given by $2n-2=4$ in the present case. One-tailed p values (probabilities for false rejection of the null hypothesis) for any pair of reaction rates were then obtained as

$$p = 1 - F(t; \nu = 4), \tag{S4}$$

where $F(t;\nu)$ is the cumulative distribution function for Student's t-distribution with ν degrees of freedom. Table S3 summarises the resulting data. p_A and p_S correspond to the metabolic rates in Figure 7 for 1250 and 2500 cells in adherent and spheroid culture, respectively. The p-values for D-Glucose and L-Lactic acid are just under 0.09 for adherent and less than 0.01 for spheroid culture, demonstrating that statistically significant differences in metabolic rates due to the addition of 700 cells can be distinguished by the micro-NMR method in both cases.

In addition, the significance of the different metabolic rates per cell (Figure 8 for adherent and spheroid culture were tested in the same way. The data is also reported in Table S3 in separate columns for cultures of 2500, 1800, and 1250 cells. In all cases, the p-values for the four metabolites reported in Fig. 8, D-Glucose, L-Lactic acid, L-Alanine, and L-Glutamine, are less than 0.005, and in all but one cases; the only exception is L-Glutamine for 1800 cells with $p_{1800} = 0.0556$.

Table S3: p-values for statistical significance of differences between metabolic rates. p_A: distinction between cultures with 2500 and 1250 adherent cells; p_S: distinction between spheroids of 2500 and 1250 cells; p_{2500}, p_{1800}, and p_{1250}: distinction between metabolic rates of spheroids and adherent cultures with the indicated cell numbers.

	Metabolite	p_A	p_S	p_{2500}	p_{1800}	p_{1250}
1	D-Glucose	0.018	0.0042	0.0012	0.0031	0.0018
2	L-Lactic acid	0.0012	0.0001	0.0006	0.0009	0.0002
3	L-Alanine	0.0082	0.3672	0.0003	0.0006	0.0001
4	L-Glutamine	0.016	0.0292	0.0008	0.0556	0.0008
5	Glutaric acid	0.0365	0.0152	0.3713	0.0057	0.0002
6	L-Leucine	0.0136	0.044	0.0033	0.239	0.0176
7	L-Valine	0.2453	0.0159	0.0434	0.0641	0.0033
8	L-Isoleucine	0.0946	0.0419	0.0406	0.1164	0.2648
9	Pyruvic acid	0.0160	0.1874	0.0177	0.0729	0.0102
10	Acetic acid	0.0075	0.0011	0.1074	0.0011	0.0033
11	Citric acid	0.1342	0.0003	0.0179	0.0061	0.0032
12	Betaine	0.2679	0.0057	0.0032	0.0047	0.0015
